# Ectodermal‐neural cortex 1 as a novel biomarker predicts poor prognosis and induces metastasis in breast cancer by promoting Wnt/β‐catenin pathway

**DOI:** 10.1111/jcmm.15520

**Published:** 2020-07-03

**Authors:** Yuhui Zhou, Xiaojiang Tang, Ligang Niu, Yang Liu, Bin Wang, Jianjun He

**Affiliations:** ^1^ Department of Breast Surgery The First Affiliated Hospital of Xi'an Jiaotong University Xi'an China

**Keywords:** breast cancer, ENC1, metastasis, overexpressed, prognosis, β‐catenin pathway

## Abstract

Breast cancer, as the most common malignancy, is the second leading cause of cancer‐related death in women. One of the kelch family member ENC1 is involved in various pathophysiologic processes. But the role of ENC1 in breast cancer has not been investigated. The present study value the feature, clinical significance and the molecular mechanisms of ENC1 in breast cancer. The expression and prognosis value of ENC1 expression among breast cancer and normal breast tissue were investigated in The Cancer Genome Atlas database and human samples. ENC1 was knockdown to explore its function in various breast cancer cell lines. Western blot was performed to explore the potential molecular mechanisms. We observed that ENC1 was overexpressed in breast cancer tissues. ENC1 overexpression was associated with high metastasis and predicted a poor prognosis in patients with breast cancer. ENC1 Knockdown inhibits the growth, clone formation, migration and invasion of breast cancer cells. Mechanism analysis revealed ENC1 was strong associated with the metastasis by modulating β‐catenin pathway. Our study emphasizes that ENC1 is a potential prognostic and metastasis‐related marker of breast cancer, and may function as a possible therapeutic target against breast cancer.

## 1 INTRODUCTION

Breast cancer, as the most common malignancy, was proved to be the second leading cause of cancer death in women. Improved detection ways and therapy have led to 5‐year survival of more than 85%, but half of patients still die because of this disease.^1^, ^2^ The early detection ways combining efficient systemic therapies have decreased the death rate of breast cancer in North America and the European Union.^3^, ^4^ However, the incidence and the mortality of this disease are still increasing due to the lack of access to advanced diagnosis and therapy in developing areas.^1^, ^5^ It is considered incurable with the currently available therapies on metastatic breast cancer.^6^ In addition, the prognosis and survival rate of metastatic breast cancer patients are poor. Based on the published data, long‐term survivors take <5% part among these patients.^7^, ^8^ Some common diagnostic and prognostic biomarkers such as human epidermal growth factor receptor (HER2), oestrogen receptor (ER) and progesterone receptor (PR) can guide therapy in breast cancer.^9^ However, these biomarkers have some disadvantages, such as unsuitability for some breast cancer patients and bias during the assessment about receptor status.^10^, ^11^ Thus, the identification of new predictive and prognostic biomarkers plays an important role in predicting and guiding the clinical treatment for this disease especially with metastatic breast cancer.

Ectodermal‐neural cortex 1 (ENC1) encodes an actin‐associated protein and is proved function as an important role during early gastrulation and the formation of the nervous system.^12^ Broad complex BTB/POZ domain‐like structure and six copies of ‘kelch motif’ repeats were two major structural elements of ENC1 protein.^13^ Previous studies indicated that ENC1 induction is necessary for the differentiation of adipocyte.^14^ Moreover, under endoplasmic reticulum stress, ENC1 could regulate the aggregation of mutant neurotoxicity Huntingtin via p62.^15^ These findings proved that ENC1 plays an critical role in maintaining the physiological functions. However, the function of ENC1 in human cancer is still indefinable. ENC1 is reported to be related to the β‐catenin pathway to contribute to colon cancer by suppressing the differentiation of colonic cells.^16^ Additionally, low ENC1 expression predicts a better prognosis among patients with ovarian cancer.^17^ However, the ENC1 gene was reported to be negatively related with the invasiveness of human pituitary null cell adenoma and oncocytoma.^18^ These controversial roles of ENC1 drive our interests into the unknown function of ENC1 in breast cancer.

The present study first verified that ENC1 was overexpressed among breast cancer tissue in comparison with normal breast tissue. ENC1 knockdown inhibited the breast cancer cells' malignant biological properties. Notably, high expression levels of ENC1 were associated with unfavourable prognosis and high metastasis in breast cancer. These findings indicated that ENC1 could be a potential therapeutic target in patients with breast cancer.

## 2 MATERIALS AND METHODS

### 2.1 The Cancer Genome Atlas database and gene expression analysis

Totally 1095 breast cancer samples and 114 normal breast samples in The Cancer Genome Atlas (TCGA) dataset were downloaded from https://xenabrowser.net/datapages/. In addition, the ENC1 expression was analysed between these two groups. In total, 107 pairs of breast cancer samples with matched normal tissues were used to analyse the ENC1 expression in paired samples. The online tool GEPIA^19^ was used to confirm the expression level of ENC1 in breast cancer and normal samples.

### 2.2 Diagnostic and prognostic analyses

The diagnostic ability of ENC1 in breast cancer was discussed with the data from TCGA database by using a receiver operating characteristic (ROC) curve drawn via SPSS 18.0 (SPSS, Inc). Cox proportional hazard regression model‐based multivariate and univariate analyses were used to explore the correlation between the clinicopathological factors and the prognosis of breast cancer patients. In addition, the Kaplan‐Meier plotter^20^ and OncoLnc^21^ were separately used to evaluate the association between the expression of ENC1 and the prognosis of patients with breast cancer (low vs high group on the basis of median ENC1 expression, which was contained in the high group).

### 2.3 Samples and immunohistochemistry

Paraffin‐embedded samples (24 breast cancer specimens with 10 lymphatic metastasis specimens and 24 non‐neoplastic breast specimens) were obtained by surgery and identified by three pathologists from the Department of Breast Surgery, The First Affiliated Hospital of XJTU School of Medicine with institutional review board approval. Informed consent was obtained from patients before the surgery. The clinicopathological characteristics, including sex, age, grade and metastasis are presented in the Table S1. For immunohistochemistry (IHC), the specimens were cut into sections (5 μm). Then, the sections were deparaffinized and rehydrated in a graded series of ethanol and washed in phosphate buffer saline. Next, they were incubated with anti‐ENC1 antibody (cat.no. sc‐517590; Santa Cruz Biotechnology, Inc) overnight (4°C) and secondary antibody (Beijing Zhongshan Jinqiao Biotechnology Co., Ltd.) for 30 minutes. For visualization, diaminobenzidine (Beijing Zhongshan Jinqiao Biotechnology Co., Ltd.) was used. Light microscope (Olympus) was used in observing and photographing the staining. The experiment was repeated in triplicate, and the integrated optical density (IOD) value was calculated in five random fields from different samples.

### 2.4 Cell culture and small interfering RNA transfection

Human breast non‐tumorigenic epithelial cell line MCF‐10A (Shanghai Cell Bank, Type Culture Collection Committee, Chinese Academy of Sciences) was cultured in Dulbecco's modified Eagle medium with 15% foetal bovine serum (FBS). Human breast cancer cell lines MDA‐MB‐231 and MCF‐7 (Shanghai Cell Bank, Type Culture Collection Committee, Chinese Academy of Sciences) were cultured in 1640 medium with 10% FBS and maintained in an incubator with 5% CO_2_ at 37°C. For transient small interfering RNA (siRNA) transfection, cells were seeded into normal growth medium at 30% confluence in 6‑well tissue plates 24 hours prior to transfection with 8 nmol/L siRNAs targeting ENC1 (control sense: 5′‐UUC UCC GAA CGU GUC ACG UTT‐3′, antisense: 5′‐ACG UGA CAC GUU CGG AGA ATT‐3′; siRNA1: ENC1 sense: 5′‐CUC UAA AGC AGG UAG AAC AdTdT‐3′, antisense: 5′‐UGU UCU ACC UGC UUU AGA GdTdT‐3′; siRNA2: ENC1 sense: 5′‐CAA UUC CAU CCA CCC AGA AdTdT‐3, antisense: 5′‐UUC UGG GUG GAU GGA AUU GdTdT‐3′) via Lipofectamine 2000 reagent (Invitrogen; Thermo Fisher Scientific, Inc), according to the manufacturer's instructions. All siRNAs and non‑specific siRNA (si‐NC) were constructed by GenePharma (Shanghai GenePharma Co., Ltd.).

### 2.5 RNA extraction and reverse transcription‑quantitative polymerase chain reaction (RT‐qPCR)

Total RNA was extracted from cell lines by using TRIzol reagent (Takara Biotechnology Co., Ltd.), and cDNA was prepared using PrimeScript RT reagent Kit (Roche Diagnostics). RT‐qPCR was performed on a CFX96 Thermal Cycler Dice™ real‐time PCR system (Bio‐Rad Laboratories, Inc) by using SYBR GREEN (BioTools Pty. Ltd.). The mRNA expression of ENC1 was normalized to 18S rRNA. Relative mRNA expression was calculated by using the 2^−ΔΔCq^ method.^22^ Each sample was run in triplicate. The primers were as follows: ENC1: F 5′‐CAC TCC GAG AAG GCG TTAG‐3′, R 5′‐CAT CGC TGA ATG CCA AAG‐3′; 18s F 5′‑CGC CGC TAG AGG TGA AAT TC‑3′, R 5′‑CTT TCG CTC TGG TCC GTC TT‑3′. All primers were constructed by GenePharma (Shanghai GenePharma Co., Ltd.).

### 2.6 Western blot analysis

Cells were lysed in pre‐chilled radio‐immunoprecipitation assay buffer with protease inhibitors (Sigma‐Aldrich; Merck KGaA). The supernatants were collected and subjected to sodium dodecyl sulphate‐polyacrylamide gel electrophoresis by using 10% gels and transferred onto polyvinylidene fluoride membranes (Roche Diagnostics). Next, the membranes were blocked with 5% non‐fat dry milk for 1.5 hours at room temperature. The membranes were then incubated with primary antibodies: anti‐ENC1 (above); anti‐glyceraldehyde‐3‐phosphate dehydrogenase (GAPDH) (Abgent Biotech Co., Ltd., Cat. No. AP7873a); anti‐E‐Cadherin (cat. no. 14472S; Cell Signaling Technology, Inc); anti‐N‐Cadherin (cat. no. 13116S; Cell Signaling Technology, Inc); anti‐β‐Catenin (cat. no. 8480S; Cell Signaling Technology, Inc); anti‐Lamin B1 (cat. no. 13435S; Cell Signaling Technology, Inc); and anti‐Vimentin (cat. no. ab92547; Abcam). Then the membranes were subjected to incubation with species‐specific horseradish peroxidase‐conjugated secondary antibodies from ZSGB‐BIO. In addition, immunoblotting signals were visualized using the Western Bright ECL detection system (Advansta, Inc).

### 2.7 Cell proliferation assay

Cells (1000 cells/well) were seeded and cultured in 96‐well plates for 1, 3, 5 and 7 days. At the indicated times, 20 μL of 3‐(4,5‐dimethyl‐2‐thiazolyl)‐2,5‐diphenyl‐2‐H‐tetrazolium bromide (MTT) (Sigma‐Aldrich; Merck KGaA) was added into the medium at a final concentration of 0.5 mg/mL and incubated for 4 hours, followed by adding 150 μL of dimethyl sulphoxide for an additional 15 minutes. A microplate reader (Dynatech Laboratories) was used to measure the absorbance by using a test wavelength of 570 nm.

### 2.8 Transwell assay

Transwell (8.0 μm pore size; Corning Inc) were used to assess cell migration and invasion. For cell invasion assay, transwell chamber was coated with Matrigel (4 × dilution; 15 μL/well; BD Biosciences). Cells were suspended in 200 μL of medium containing 0.5% FBS and seeded in the upper chamber with a density of about 3‐9 × 10^4^ cells/mL for migration assay and 1 × 10^5^ cells/mL for invasion assay. Medium with 20% FBS (1 mL) was added to the lower chamber. Non‐migrating/non‐invading cells in the upper chamber were removed with a cotton swab after a 12 or 24 hours incubation. Then, migrating/invading cells were fixed with 100% methanol followed by staining with crystal violet solution (0.5% crystal violet in 2% ethanol). The images were captured with an inverted Olympus IX71 microscope (Olympus Corp.). The migrated cells were calculated in three random fields.

### 2.9 Bioinformatic analysis

The co‐expressed genes related with ENC1 were screened from Coexpedia (http://www.coexpedia.org/).^23^ In addition, the top 40 genes were analysed as a group to find the potentially involved biological processes with FunRich 2.1.2 software to explore the detail mechanisms of ENC1 in breast cancer.

### 2.10 Statistical analysis

Each assay was performed and calculated in triplicate (N = 3). Two‐way ANOVA with Bonferroni post‐test was used for MTT groups’ comparisons. One‐way ANOVA with Dunnett's post‐test was performed in multiple groups’ comparisons. *P* < 0.05 was considered statistically significant. The results are shown as the mean ± standard deviation. The Kaplan‐Meier survival curve was used to assess the survival of patients with breast cancer. Univariate and multivariate Cox regression analyses were used to evaluate the effects of different ENC1 expression on different clinic pathological Factors. Calculations and graphing were performed with SPSS 18.0 (SPSS Software, Inc) and GraphPad Prism 5 (GraphPad Software, Inc).

## 3 RESULT

### 3.1 ENC1 was overexpressed in breast cancer

**FIGURE 1:**
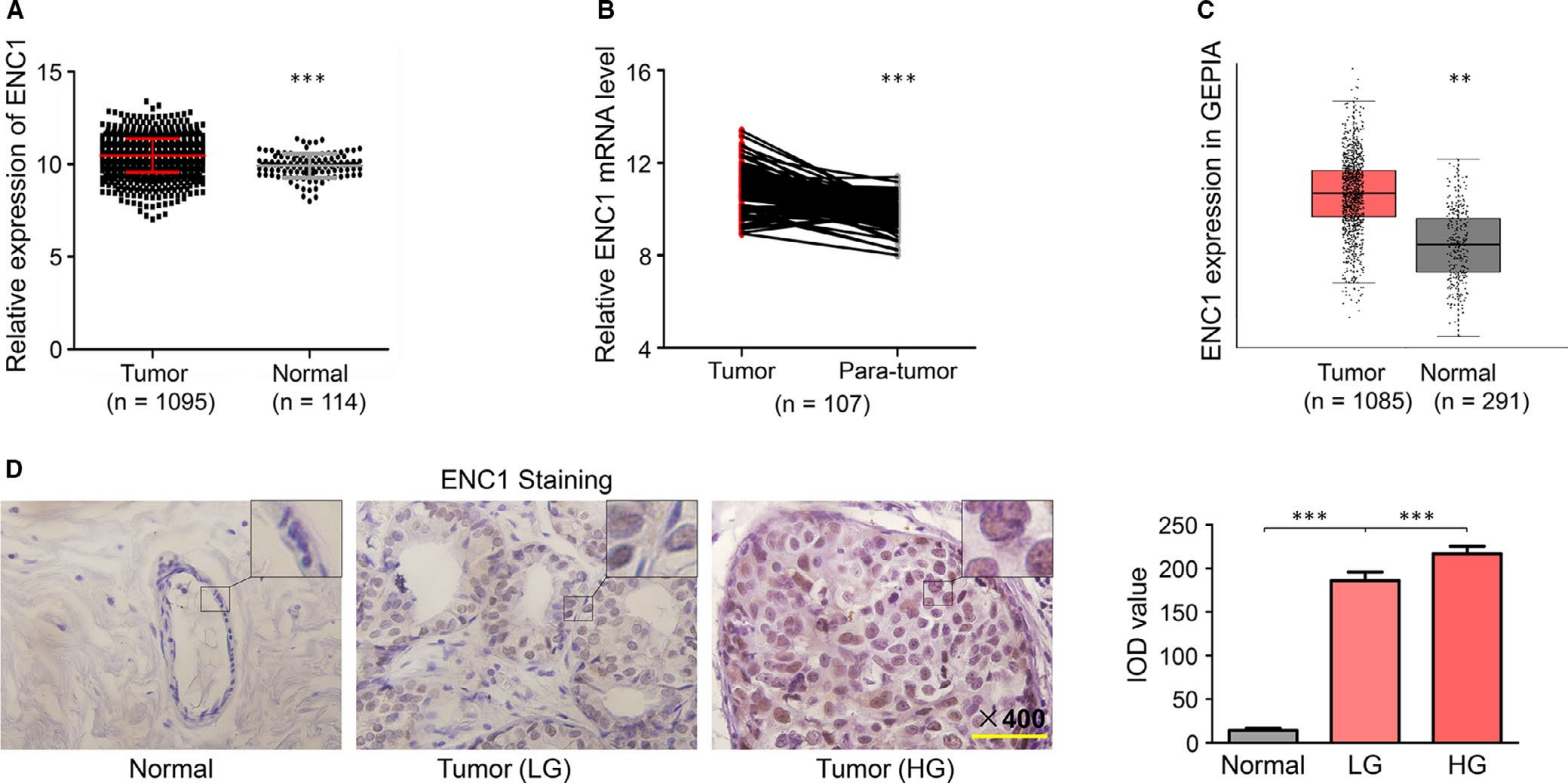
ENC1 expression is increased in breast cancer. A, ENC1 expression in breast cancer (tumour) and normal breast tissues (normal) in TCGA dataset. B, ENC1 gene expression was significantly higher in breast cancer (tumour) compared with matched non‑neoplastic breast tissues (para‐tumour) in TCGA dataset. C, ENC1 expression in breast cancer (tumour) and normal breast tissues (normal) in GEPIA online tools. Immunohistochemical staining was performed to detect ENC1 expression in breast cancer with different grades (low grade: LG, high grade: HG) and normal breast specimens. Representative ENC1 staining was shown, and IOD value was used to quantify the results. The experiments were performed and calculated in triplicate (N = 3). Scale bars, 200 µm. (D)

ENC1 expression between normal breast tissues and breast cancer tissues in TCGA dataset was investigated. In Figure 1A, the expression of ENC1 mRNA was significantly higher in tumour tissues in comparison with non‐tumour tissues. Moreover, the ENC1 expression level in breast cancer was significantly higher compared with it in matched normal breast tissues (Figure 1B). These results were also supported by the data analysed in GEPIA (Figure 1C). For further confirming the ENC1 expression panel in breast cancer, IHC was performed with our samples. As can be seen in Figure 1D, the ENC1 staining was stronger in breast cancer compared with normal breast. Additionally, the ENC1 staining in high‐grade breast cancer (HG) was much stronger than that in low‐grade breast cancer (LG). Collectively, these results indicated an oncogenic role of ENC1 in breast cancer.

### 3.2 Increased expression of ENC1 is a potential diagnostic marker and associated with poor prognosis among patients with breast cancer

**FIGURE 2:**
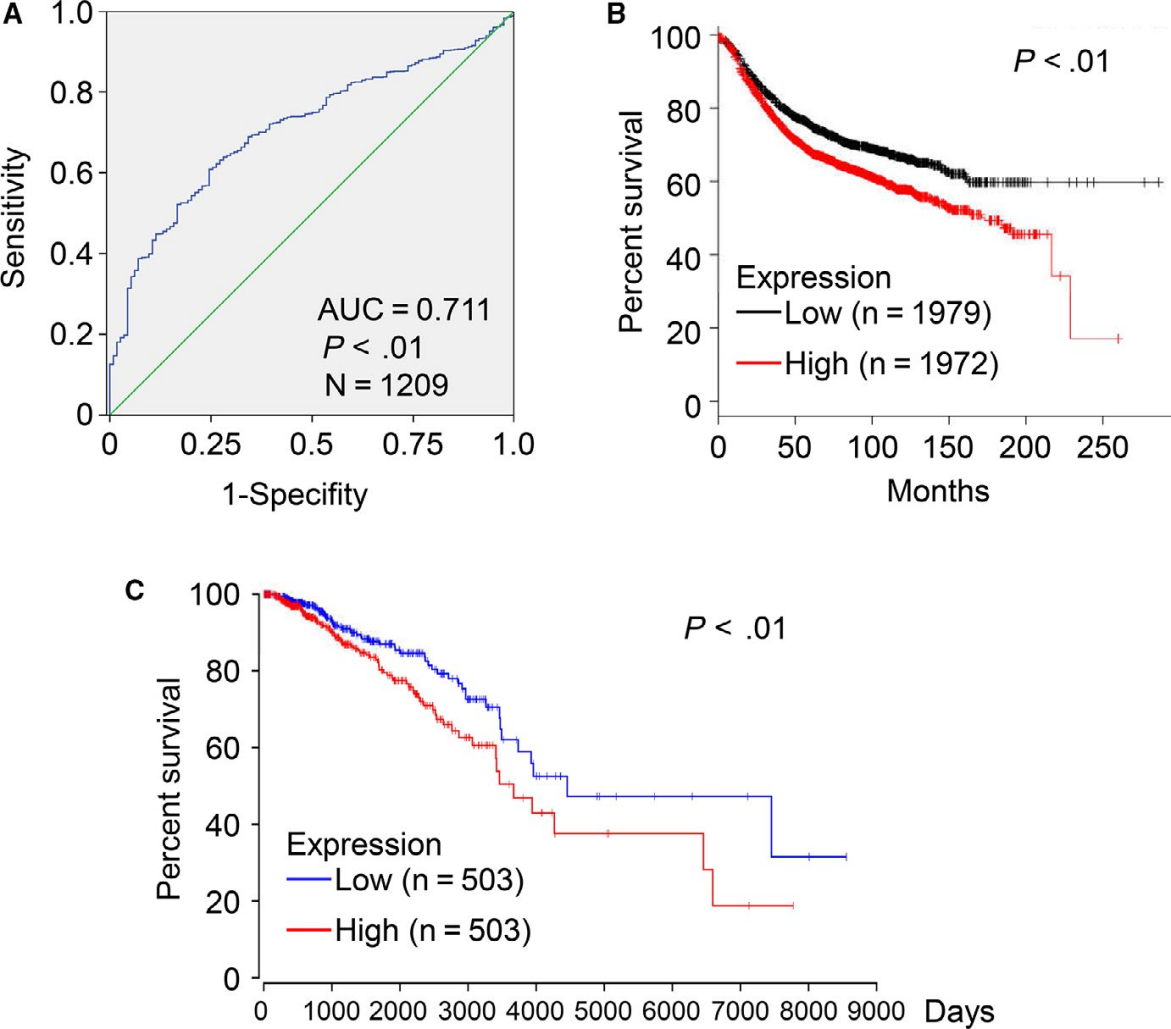
Diagnosis and prognostic value of ENC1 in breast cancer. (A) ROC curve of ENC1 expression in breast cancer. The curve indicated that ENC1 possessed a moderate diagnostic ability for breast cancer (AUC = 0.711; *P* < 0.01). The result from Kaplan‐Meier plotter (B) and OncoLnc (C) demonstrated that high ENC1 expression was significantly associated with a reduced overall survival time (*P* < 0.01)

To determine whether ENC1 can function as a diagnostic marker of breast cancer, an ROC curve was drawn with TCGA data. The AUC value is 0.711, revealed the moderate diagnostic value of ENC1 in breast cancer (*P* < 0.01, Figure 2A). Moreover, the overall survival (OS) time of patients with breast cancer was analysed by both Kaplan‐Meier plotter (Figure 2B) and OncoLnc (Figure 2C). The results indicated that high ENC1 expression (median as the cut‐off) was associated with wore prognosis among breast cancer patients. Next, analysis by both univariate and multivariate cox regression revealed that the OS time of breast cancer patients was also associated with ENC1 expression except for lymph node metastasis and distant metastasis in TCGA database (Table 1). Besides, chi‐square test was performed to investigate the association between the ENC1 expression level and different clinicopathological variables. Notably, high ENC1 expression was significantly predicted the lymph and distant metastasis, and high ENC1 expression may predict negative expression of ER, PR and HER2 though only PR shows the statistic difference (Table 2). These results may due to the complex molecular changes in different individuals that may influence the function of ENC1.^24^ Altogether, these data demonstrated that ENC1 had a diagnostic accuracy for breast cancer patients and also supported the potential value of ENC1 in breast cancer metastasis and prognosis.

**TABLE 1:** Prognostic value of clinicopathological factors and *enc1* overexpression using univariate and multivariate cox regression analysis (n = 603)

Variate	Univariate analysis		Multivariate analysis	
	Hazard ratio (95% CI)	*P*‐value	Hazard ratio (95% CI)	*P*‐value
Clinical stage (I/II‐IV)	2.12 (0.84‐5.38)	0.114	1.56 (0.55‐4.42)	0.399
Age (≥50/<50)	2.31 (0.88‐3.48)	0.114	1.56 (0.77‐3.16)	0.214
Lymph metastasis (yes/no)	1.89 (1.02‐3.55)	0.048	1.43 (1.01‐2.98)	0.049
Distant metastasis (yes/no)	6.21 (2.73‐14.09)	<0.001	4.94 (1.99‐12.24)	0.001
ENC1 expression (high/low)	0.92 (1.21‐1.76)	0.013	0.69 (1.16‐1.31)	0.037

**TABLE 2:** Correlation between ENC1 expression and clinicopathological variables in patients with breast cancer (n = 603)

Variable	Number	ENC1 expression		χ^2^‐test
		Low	High	*P*‐value
Age (y)				
≥50	438	218	220	0.607
<50	165	86	79	
Lymph metastasis				
Yes	314	137	177	0.001
No	289	167	122	
Distant metastasis				
Yes	13	3	10	0.046
No	590	301	289	
Clinical stage				
II‐IV	483	240	243	0.475
I	120	64	56	
Oestrogen receptor				
Positive	232	121	111	0.274
Negative	371	128	143	
Progesterone receptor				
Positive	226	126	100	0.0264
Negative	377	175	202	
HER2				
Positive	407	208	199	0.233
Negative	196	90	106	

### 3.3 ENC1 enhances the proliferation properties of breast cancer cells

**FIGURE 3:**
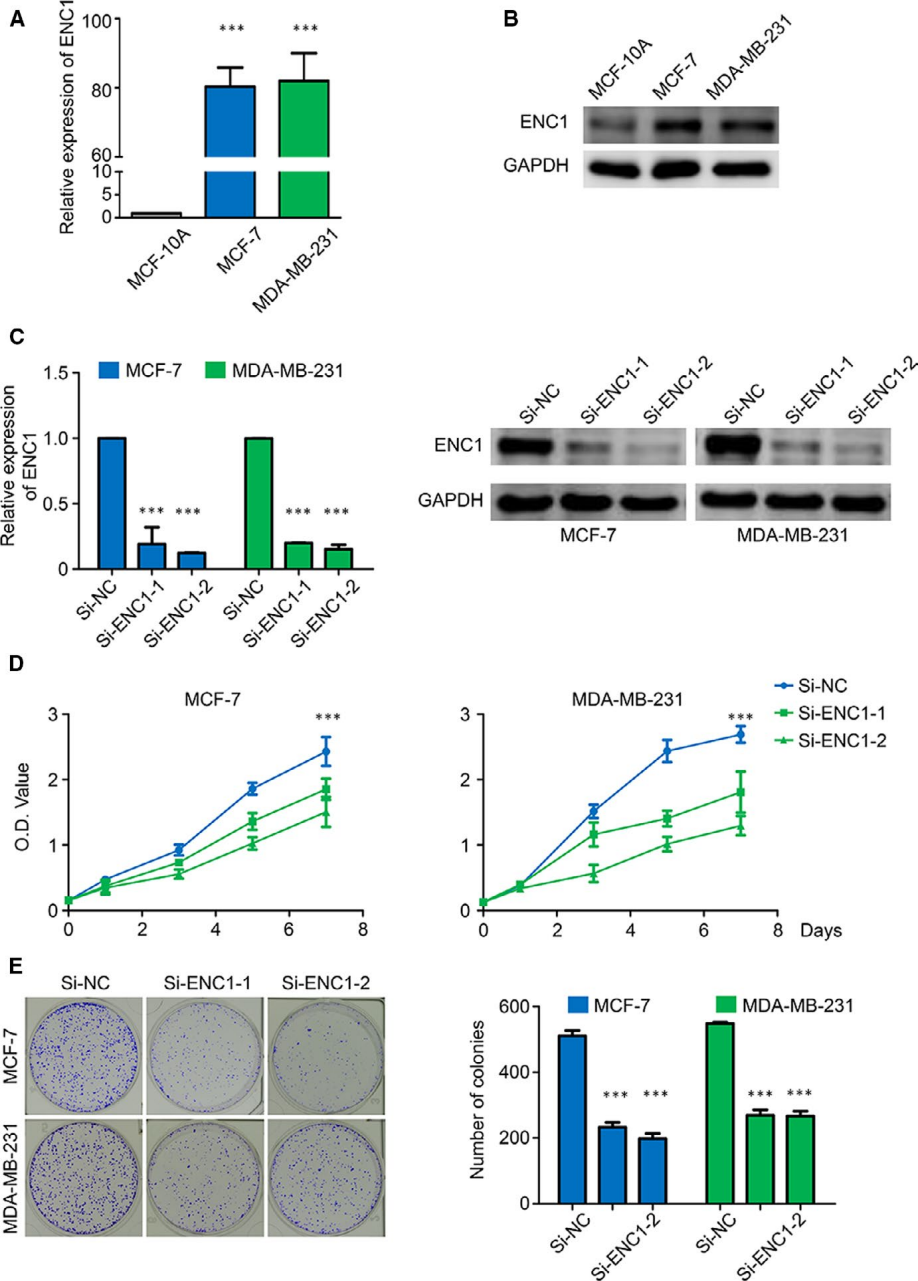
ENC1 enhances the proliferation properties of breast cancer cells. The ENC1 expression in different cell lines was demonstrated by qRT‐PCR (A) and Western blot analysis (B). Knockdown of ENC1 mRNA with two different siRNAs (si‐ENC1‐1 and si‐ENC1‐2) in MCF‐7 and MDA‐MB‐231 cells was demonstrated by RT‐qPCR and Western blot analysis. The 18S RNA was used as a normalized control for RT‑qPCR assay, and GAPDH was utilized as a loading control for Western blot analysis (C). (D) ENC1 knockdown significantly inhibited cell viability. (E) ENC1 knockdown significantly inhibited colony formation of breast cancer cells. The representative images of colony formation in cells transfected with the indicated siRNAs are shown. The experiment is repeated and calculated in triplicate (N = 3). Data are presented as mean ± standard deviation. ***P < 0.001

Given that the expression of ENC1 was higher in breast cancer cell lines in comparison with breast non‐tumorigenic cell line (Figure 3A,B), we performed knockdown experiments in breast cancer cell lines MCF‐7 and MDA‐MB‐231 to illustrate the malignant biological function of ENC1. ENC1 knockdown by two siRNAs with different sequences (si‐ENC1‐1 and ‐2) was confirmed both at mRNA level with RT‐qPCR and at the protein level by Western blot analysis (Figure 3C). Further experiments show ENC1 knockdown inhibited cell proliferation (Figure 3D) and colony formation (Figure 3E) of both breast cancer cell lines.

### 3.4 ENC1 strengthens the metastasis properties of breast cancer cells

**FIGURE 4:**
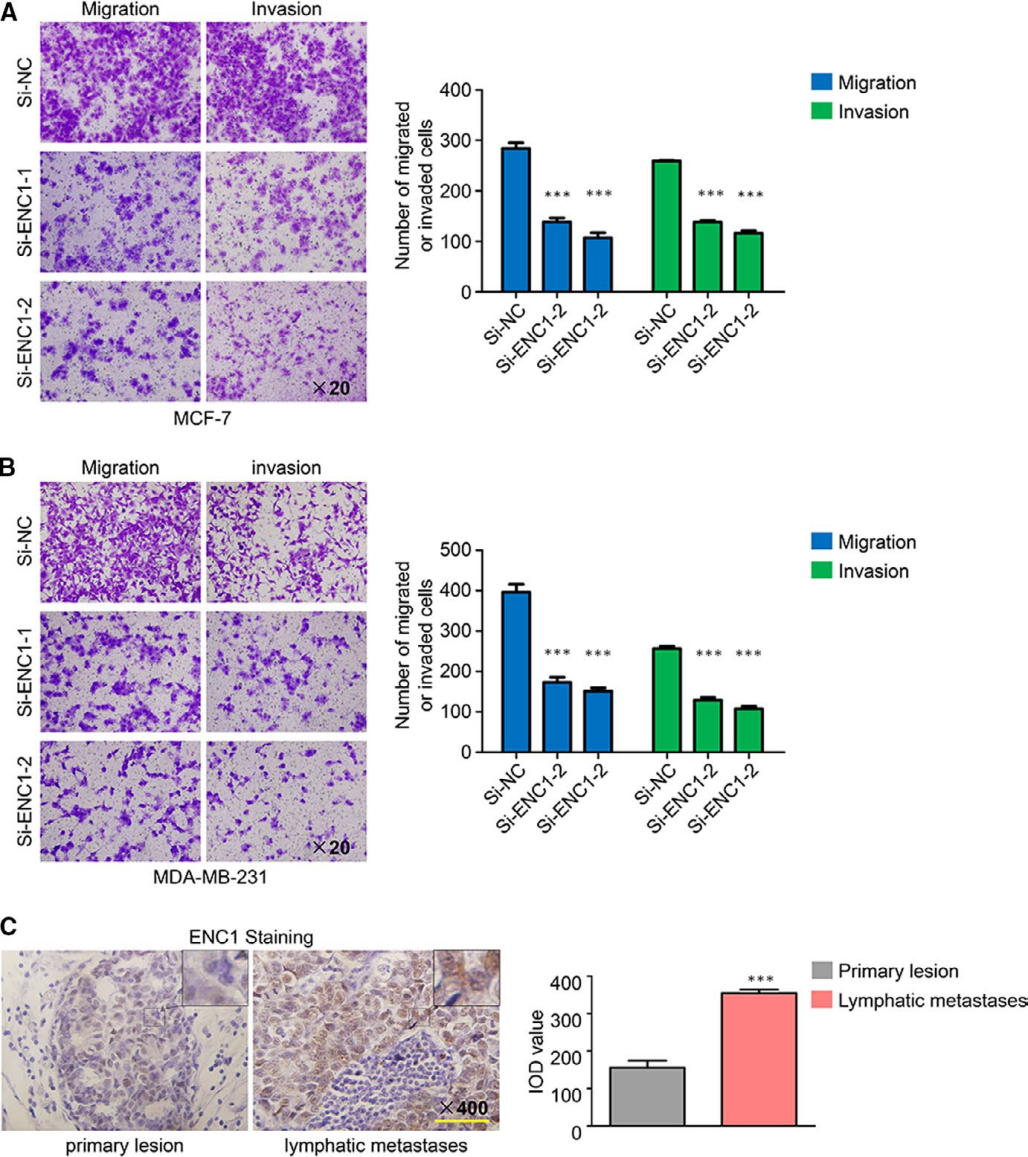
ENC1 enhances the metastasis properties of breast cancer cells. (A, B) Effects of ENC1 knockdown on migration and invasion of both cell lines were measured by transwell assays. Represent fields are shown. (C) Immunohistochemical staining was performed to detect ENC1 expression in the primary lesion and the lymphatic metastasis lesion of a patient with breast cancer. Integrated optical density (IOD) value was used to quantify the results. The experiment is repeated and calculated in triplicate (N = 3). Scale bars, 200 µm. Data are presented as mean ± standard deviation. ***P < 0.001

Given that the analysis above verified that ENC1 was associated with breast cancer metastasis, then we explored the role of ENC1 in cancer‐associated mortality by using transwell assay. As can be shown in Figure 4A,B, the number of migrated and invaded cells was significantly lower in the si‐ENC1 transfected groups than that in the si‐NC‐transfected groups. Then we performed IHC by using the primary lesion and the lymphatic metastasis lesion of the same breast cancer patient sample. As can be seen in Figure 4C, the ENC1 staining in lymphatic metastasis lesion was much stronger than that in the primary lesion. These results demonstrated that ENC1 had supported the breast cancer metastasis.

### 3.5 Increased expression of ENC1 enhanced metastasis andβ‐catenin pathway in breast cancer cells

**FIGURE 5:**
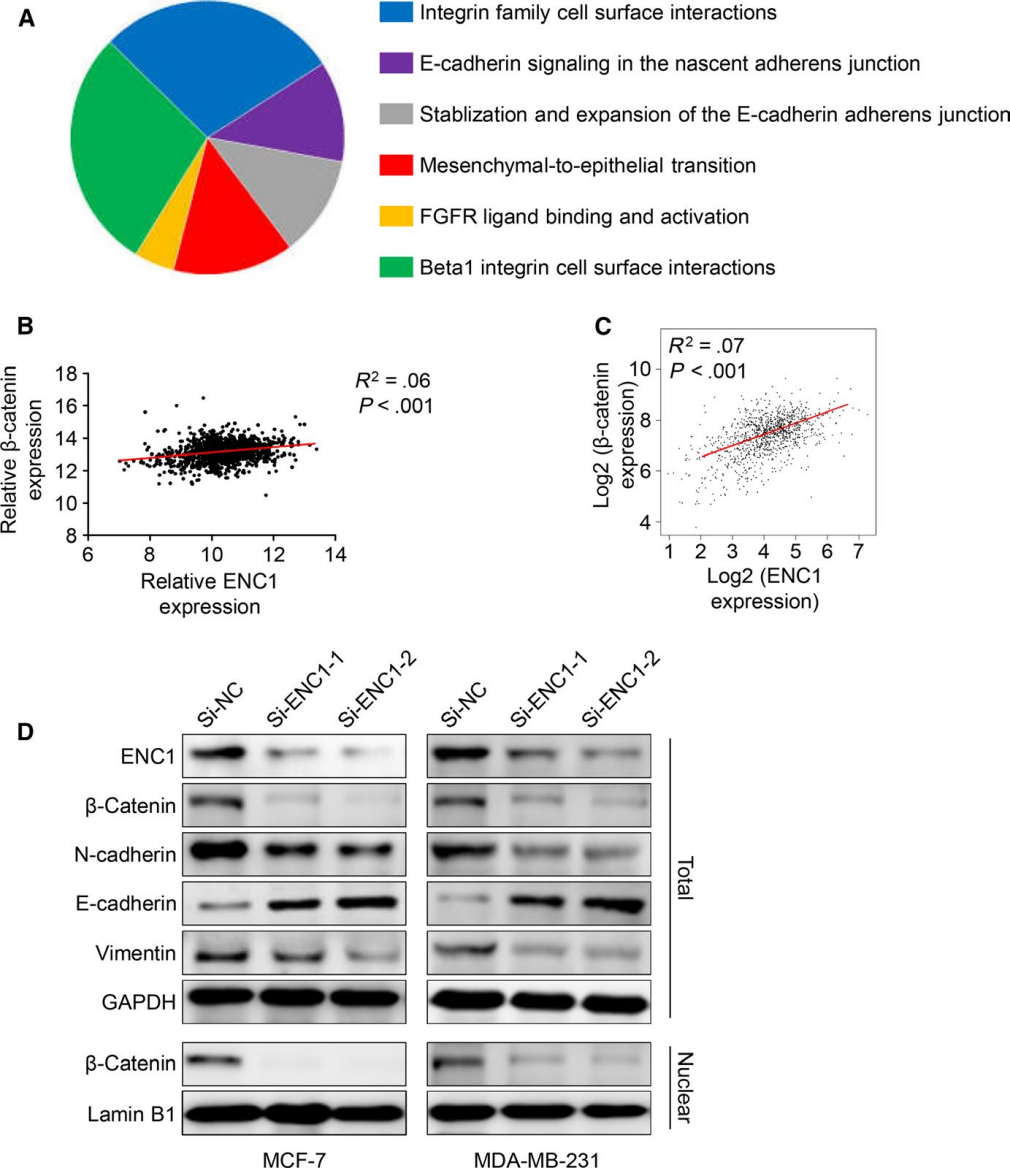
ENC1 induced metastasis by promote β‐catenin pathway. (A) Potential ENC1‐related biological pathways in breast tissues were identified by FunRich. The expression of ENC1 and β‐catenin was positively correlated both in TCGA database (B) and GEPIA (C). (D) Western blot analysis the effect of ENC1 knockdown on β‐catenin pathway and different cell adhesion‐associated molecules

To further clarify the mechanism underlying the tumour‐promoting effects of ENC1 in breast cancer, a set of ENC1 neighboured genes which were related to ENC1 in the breast cancer were searched from Coexpedia. Then, the biological processes of these group genes were investigated using FunRich. The result demonstrated that ENC1 was strongly related to cell interaction and cell adhesion in breast tissues (Figure 5A). These results were consistent with our above findings that the aberrant expression of ENC1 will lead to the high metastasis in breast cancer. As previous study supported that ENC1 may function as an oncogene by mediate Wnt/β‐catenin pathway in colorectal carcinomas.^16^ We thus suspect ENC1 may also promote breast cancer though Wnt/β‐catenin pathway. Consist with our hypothesis, the expression of ENC1 and β‐catenin was positively correlated in breast cancer among TCGA database (Figure 5B) and GEPIA (Figure 5C). Moreover, down‐regulation of ENC1 obviously decreased both total and nucleus β‐catenin expression (Figure 5D). Besides, ENC1 knockdown increases the expression level of epithelial cell marker E‐cadherin while reducing the level of mesenchymal marker N‐cadherin and vimentin (Figure 5D). Altogether, these results indicated that ENC1 may enhance metastasis by mediating β‐catenin pathway in breast cancer.

## 4 DISCUSSION

Improvements in early‐stage diagnosis and development of therapeutic targets have improved the OS time in breast cancer patients. However, several individuals still suffer from poor survival rate due to high metastasis of breast cancer.^25^, ^26^ Fortunately, many recent studies about breast cancer have focused on this issue.^27^, ^28^ However, as the incidence rate of breast cancer worldwide is still high, the novel biomarkers and new strategies are urgently needed.^9^ Therefore, the exploration of new biomarkers in breast cancer is of great importance.

In the present study, we first discovered that ENC1 was up‐regulated among breast cancer compared with normal breast tissues. Next, we analysed the different datasets and identified ENC1 was a special prognosis and metastasis biomarker of breast cancer. Analysis of TCGA database revealed the high level of ENC1 expression, indicating the poor prognosis and high metastasis in patients with breast cancer. We supposed that these conditions may increase the therapeutic value of ENC1. We also found knocking down ENC1 in breast cancer will decrease the proliferation, migration, invasion and colony formation of breast cancer cells. As for further exploring the mechanism of ENC1 in promoting breast tumorigenesis, the co‐expression genes of ENC1 were investigated via Coexpedia. Totally 275 genes were identified. Then, the top 40 genes were analysed as a group to search the potential function of ENC1 in breast cancer. Consistently, these molecular portraits of ENC1 revealed its strong association with metastasis‐related pathways. These findings were further confirmed by correlation analysis, IHC staining and transwell experiment. Collectively, these results suggested ENC1an important role in breast cancer development, especially cancer cell metastasis. Thus, we supposed that ENC1 could be a possible therapeutic target among invasive breast cancer.

Initially, ENC1 was most abundant in foetal brain, which is correlated with the development of the nervous system; it is also expressed in the foetal kidney and liver, but its activity is reduced when the organs came into adult stage.^13^, ^29^ Thus, ENC1 may play a determined role in the differentiation of a variety of cell lineages. This finding is in line with our result, indicating that ENC1 is associated with breast cancer metastasis. Moreover, univariate and multivariate cox regressions reflected that ENC1 expression is a significant prognostic factor. We suppose that the complex molecular background or special gene mutation in different individuals may also take charge of it. In addition, further study towards the relationship between ENC1 and breast cancer cell special molecular such as HER2 and BRCA1 is essential. Previous studies that focused on ENC1 function in different human cancers have revealed the controversial role of ENC1.^16^, ^17^, ^30^ The present study found that high ENC1 expression caused the high metastasis abilities of breast cancer cells, which was consistent with the finding that ENC1 was associated with invasiveness of both pituitary null cell adenoma and oncocytoma.^18^ Given that ENC1 could up‐regulate the β‐catenin in colorectal carcinomas and that β‐catenin pathway regulates the metastasis of breast cancer,^16^, ^31^ we thus have been suggested that ENC1 may promote breast cancer metastasis via up‐regulating the β‐catenin pathway. Our finding that ENC1 staining is stronger in lymph metastasis tissues and knockdown of ENC1 is inhibited the β‐catenin expression supported our hypothesis. Interestingly, a putative membrane‐associated progesterone steroid receptor PGRMC1 was recently proved to be able to active β‐catenin pathway in lung adenocarcinoma.^32^ And our finding ENC1 expression predicting negative expression of PR indicates the potential negative feedback between β‐catenin pathway and progesterone steroid receptor. Thus, ENC1 negatively regulated the progesterone steroid receptor may through β‐catenin pathway. However, further investigations into the detail molecular mechanisms are still needed to confirm this hypothesis. In addition, we simply used the data from the database to evaluate the prognosis of breast cancer patients because we did not collect enough patient data. These factors are a limitation of the present study.

Altogether, ENC1 is significantly overexpressed among breast cancer and had the moderately diagnostic and prognostic value in breast cancer. Importantly, high ENC1 expression was associated with high metastasis in breast cancer. ENC1 is supposed to become another novel diagnostic, metastatic and prognostic biomarker even a target for breast cancer in the future.

## CONFLICT OF INTEREST

The authors declare that they have no competing interests.

## AUTHOR CONTRIBUTION

**Yuhui Zhou:** Data curation (lead); Formal analysis (lead); Investigation (lead); Methodology (lead); Writing‐original draft (lead); Writing‐review & editing (lead). **Xiaojiang Tang:** Data curation (equal); Investigation (equal). **Ligang Niu:** Project administration (equal). **Yang Liu:** Methodology (equal). **Bin Wang:** Funding acquisition (equal); Resources (equal). **JianJun He:** Conceptualization (lead); Resources (lead); Supervision (lead); Writing‐original draft (equal); Writing‐review & editing (equal).

## ETHICAL APPROVAL

All the patients have signed the informed consent before surgery. Ethics Committee of the First Affiliated Hospital of Xi'an Jiaotong University approved this study.

## Supporting information

Table S1

## Data Availability

The datasets used during the current study are available from the corresponding author on reasonable request.
