# Supplementary material for: Ectodermal‐neural cortex 1 as a novel biomarker predicts poor prognosis and induces metastasis in breast cancer by promoting Wnt/β‐catenin pathway
Source: J Cell Mol Med. 2020 Jul 3;24(15):8826–35. doi: 10.1111/jcmm.15520 (PMC7412682; doi:10.1111/jcmm.15520)
Supplement: Supplementary file 1 — Table S1 [file JCMM-24-8826-s001.docx]

**Supplementary Table I. Clinicopathological characteristics of the breast cancer patients (n=24)**

| **Characteristics** | **No.** | **Percent** |
| --- | --- | --- |
| Gender |  |  |
| Male | 0 | 0 |
| Female | 24 | 100 |
| Age, years |  |  |
| Mean | 48.5 |  |
| SD | 13.4 |  |
| grade |  |  |
| I | 8 | 33.3 |
| II /III/IV | 16 | 66.7 |
| lymphatic metastasis |  |  |
| No | 14 | 58.3 |
| Yes | 10 | 41.7 |
| distant metastasis |  |  |
| No | 23 | 95.8 |
| Yes | 1 | 4.2 |
